# Supplementary material for: Galanin receptor 1 expressing neurons in hippocampal-prefrontal circuitry modulate goal directed attention and impulse control
Source: bioRxiv. 2025 Aug 9:2024.07.29.605653. Originally published 2024 Jul 29. Preprint. [Version 3] doi: 10.1101/2024.07.29.605653 (PMC11312591; doi:10.1101/2024.07.29.605653)
Supplement: Supplement 1 [file NIHPP2024.07.29.605653v3-supplement-1.pdf]

bioRxiv preprint doi: https://doi.org/10.1101/2024.07.29.605653; this version posted August 9, 2025. The copyright holder for this preprint (which was not certified by peer review) is the author/funder. This article is a US Government work. It is not subject to copyright under 17 USC 105 and is also made available for use under a CC0 license.

**Supplementary Figure 1. Expression of galanin fibers in the vPFC and the vHC.**

**(a)** Schematic representing the location of the area analyzed in the PFC. **(b)** Microphotograph shows the distribution of galanin fibers and terminals in the PFC subdivisions (scale bar: 200 µm). **(c)** Magnified image of the galanin-containing fibers and terminals in the IL (scale bar: 200 µm). **(d)** Quantification of the mean density of galanin immunofluorescence in the three PFC subdivisions (Cg1: 0.43 ± 0.035, PrL: 0.43 ± 0.04, IL: 0.47 ± 0.04; au: arbitrary unit; N = 5 animals, 15 sections per region). Bar chart represents mean ± SEM. Dots represent individual animals. **(e)** Schematic representing the location of the area analyzed in the HC. **(f)** Microphotograph shows the distribution of galanin fibers and terminals in the vHC subdivisions (scale bar: 200 µm). **(g)** Magnified image of the galanin-containing fibers and terminals in the vCA1 (scale bar: 200 µm). **(h)** Quantification of the mean density of galanin immunofluorescence in the vHC subdivisions (CA1: 2.32 ± 0.22, CA2: 2;51 ± 0.10, CA3: 2.06 ± 0.11, DG: 2.40 ± 0.18; au: arbitrary unit; N = 1 animal, 4 to 15 sections per region). Bar chart represents mean ± SEM. Dots represent individual animals.

**Supplementary Figure 2. Statistics for the main behavioral outcomes assessed in the 5-choice task for the optogenetic experiment.**

bioRxiv preprint doi: https://doi.org/10.1101/2024.07.29.605653; this version posted August 9, 2025. The copyright holder for this preprint (which was not certified by peer review) is the author/funder. This article is a US Government work. It is not subject to copyright under 17 USC 105 and is also made available for use under a CC0 license.

**Supplementary Figure 3. Statistics for the latency measures in the 5-choice task for the optogenetic experiment.**

**Supplementary Figure 4. Proportion of response types between laser conditions.**

**(a)** Proportion of response types in non-stimulated and stimulated trials for vPFC-ChR2 animals (rats n = 8). **(b)** Proportion of response types in non-stimulated and stimulated trials for vPFC-tdTomato animals (rats n = 7). **(c)** Proportion of response types in non-stimulated and stimulated trials for vHC-ChR2 animals (rats n = 12). **(d)** Proportion of response types in non-stimulated and stimulated trials for vHC-tdTomato animals (rats n = 5).

**Supplementary Figure 5. Comparison of MO and PrL/IL optical stimulation on behavioral responses.**

**(a)** Schematic of brain section showing location of viral expression in the medial orbital region (MO) with corresponding photomicrograph. **(b)** Proportion of response types in non-stimulated and stimulated trials for animals with optic fiber in the MO brain region (rats n = 8). **(c-f)** Behavioral effects of optical stimulation for MO or PL/IL GalR1-expressing cells on accuracy, omissions, premature responses, and correct response latencies (MO: n = 8; IL: n= 8). Error bars represent SEM. * $p < 0.05$ (paired t-test between conditions after significant interaction effect in mixed ANOVA)

**Supplementary Figure 6. Location of optical fibers in the vPFC and vHC for the optogenetics experiments.**

bioRxiv preprint doi: https://doi.org/10.1101/2024.07.29.605653; this version posted August 9, 2025. The copyright holder for this preprint (which was not certified by peer review) is the author/funder. This article is a US Government work. It is not subject to copyright under 17 USC 105 and is also made available for use under a CC0 license.

**Supplementary Figure 7. Location of optical fibers in the vPFC and vHC for the photometry experiments.**

# Figure S1

## Expression of galanin fibers in vPFC and vHC

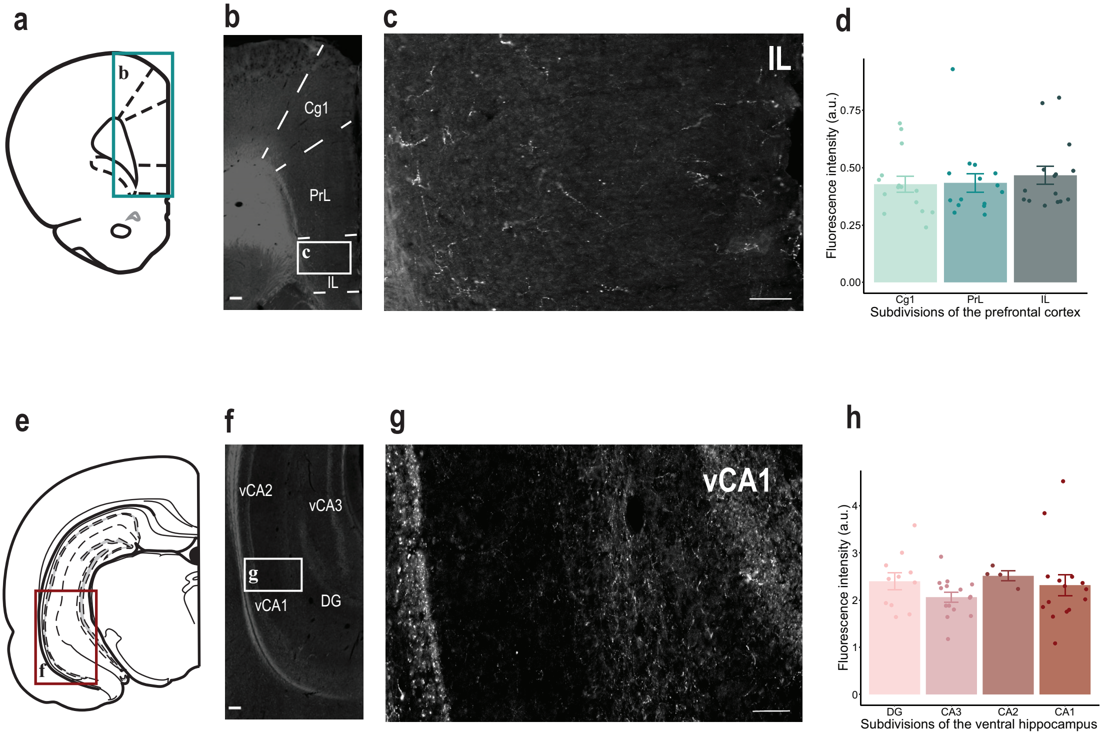

bioRxiv preprint doi: https://doi.org/10.1101/2024.07.29.605653; this version posted August 9, 2025. The copyright holder for this preprint (which was not certified by peer review) is the author/funder. This article is a US Government work. It is not subject to copyright under 17 USC 105 and is also made available for use under a CC0 license.

# Figure S2

**Statistics of the parameters measured in the 5-choice task optogenetic experiments**

| | | Accuracy | Correct response | Incorrect response | Omission | Premature response |
|---|---|---|---|---|---|---|
| **ChR2 in vPFC** | | | | | | |
| Laser OFF | Mean | **82.02081** | **51.45122354** | **10.98895492** | **17.64606213** | **19.91375941** |
| | SEM | 2.901515 | 5.53019645 | 2.003617865 | 5.105644181 | 5.989359496 |
| Laser ON | Mean | **68.98836** | **35.98660714** | **15.28822761** | **43.48285623** | **5.242309013** |
| | SEM | 4.986862 | 7.77719399 | 3.375468469 | 10.38658831 | 2.015927265 |
| **tdTomato in vPFC** | | | | | | |
| Laser OFF | Mean | **89.21182** | **63.4662332** | **7.358951268** | **15.35178713** | **13.8230284** |
| | SEM | 2.637418 | 5.568235637 | 1.798810977 | 5.2688695 | 3.921215118 |
| Laser ON | Mean | **91.90784** | **65.16947289** | **5.376452789** | **19.07976087** | **10.37431346** |
| | SEM | 1.769553 | 5.277246771 | 1.187370978 | 4.452511768 | 3.314086832 |
| Statistical comparison between groups (ChR2 and tdTomto) and conditions (Laser): (Mixed ANOVA, interaction) | | | | | | |
| | F value | 8.104 | 2.835 | 2.727 | 7.248 | 2.598 |
| | p value | 0.014 | 0.116 | 0.123 | 0.018 | 0.123 |
| | $\eta_p^2$ | 0.384 | 0.179 | 0.173 | 0.358 | 0.167 |

| | | Accuracy | Correct response | Incorrect response | Omission | Premature response |
|---|---|---|---|---|---|---|
| ChR2 in MO | | | | | | |
| Laser OFF | Mean | **89.1865** | **59.67896893** | **6.826730244** | **14.52529734** | **18.96900349** |
| | SEM | 2.637418 | 5.307878602 | 1.421312652 | 4.927990041 | 4.10098683 |
| Laser ON | Mean | **72.94635** | **45.88361308** | **12.02889976** | **17.25603009** | **24.83145707** |
| | SEM | 7.475957 | 9.682159026 | 2.441670829 | 5.310827873 | 7.779375757 |
| Statistical comparison between groups (PFC and MO) and conditions (Laser): (Mixed ANOVA, interaction) | | | | | | |
| | F value | 0.362 | 0.032 | 0.553 | 6.369 | 7.44 |
| | p value | 0.557 | 0.861 | 0.469 | 0.024 | 0.016 |
| | $\eta_p^2$ | 0.025 | 0.002 | 0.038 | 0.313 | 0.347 |
| Statistical comparison between groups (MO and tdTomato) and conditions (Laser): (Mixed ANOVA, interaction) | | | | | | |
| | F value | 10.592 | 3.855 | 8.878 | 0.078 | 3.509 |
| | p value | 0.006 | 0.71 | 0.011 | 0.784 | 0.084 |
| | $\eta_p^2$ | 0.449 | 0.229 | 0.406 | 0.006 | 0.213 |

| | | Accuracy | Correct response | Incorrect response | Omission | Premature response |
|---|---|---|---|---|---|---|
| ChR2 in vHC | | | | | | |
| Laser OFF | Mean | **85.5132** | **57.70791888** | **11.09966969** | **15.95211515** | **15.24029629** |
| | SEM | 2.90706 | 3.392341087 | 1.876534431 | 2.452586439 | 3.240376983 |
| Laser ON | Mean | **79.16721** | **51.86716637** | **12.37572558** | **25.74606523** | **10.01104282** |
| | SEM | 3.49986 | 4.445038384 | 1.550673059 | 3.175536439 | 2.263969261 |
| tdTomato in vHC | | | | | | |
| Laser OFF | Mean | **90.70627** | **69.60419998** | **7.182922929** | **11.23568916** | **11.97718793** |
| | SEM | 1.370147 | 2.739733298 | 1.190402456 | 3.272632679 | 0.927973488 |
| Laser ON | Mean | **87.83151** | **67.25955986** | **9.328444508** | **7.462850442** | **15.94914519** |
| | SEM | 2.05587 | 1.635296674 | 1.57792892 | 3.105930942 | 3.112960774 |
| Statistical comparison between groups (ChR2 and tdTomto) and conditions (Laser): (Mixed ANOVA, interaction) | | | | | | |
| | F value | 0.023 | 0.303 | 0.04 | 6.672 | 1.939 |
| | p value | 0.882 | 0.59 | 0.844 | 0.021 | 0.184 |
| | $\eta_p^2$ | 0.002 | 0.02 | 0.003 | 0.308 | 0.114 |

bioRxiv preprint doi: https://doi.org/10.1101/2024.07.29.605653; this version posted August 9, 2025. The copyright holder for this preprint (which was not certified by peer review) is the author/funder. This article is a US Government work. It is not subject to copyright under 17 USC 105 and is also made available for use under a CC0 license.

# Figure S3

**Statistics of the latencies measured in the 5-choice task optogenetic experiments**

| | | Correct response latency | Incorrect response latency | Premature response latency | Magazine latency |
|---|---|---|---|---|---|
| **ChR2 in vPFC** | | | | | |
| Laser OFF | Mean | **0.8365** | **1.942375** | **3.98875** | **1.189375** |
| | SEM | 0.044755686 | 0.215749353 | 0.200171333 | 0.068890425 |
| Laser ON | Mean | **1.15225** | **3.1695** | **3.6152** | **1.2925** |
| | SEM | 0.141407561 | 0.415298946 | 0.228427696 | 0.082934785 |
| | | | | | |
| **tdTomato in vPFC** | | | | | |
| Laser OFF | Mean | **0.848857143** | **1.747** | **4.165571429** | **1.118428571** |
| | SEM | 0.07374132 | 0.539940164 | 0.161744486 | 0.055153836 |
| Laser ON | Mean | **0.801857143** | **1.937666667** | **4.033833333** | **1.162142857** |
| | SEM | 0.06668277 | 0.403783865 | 0.174455793 | 0.061122605 |
| | | | | | |
| Statistical comparison between group and conditions (Mixed ANOVA, interaction) | | | | | |
| | F value | 8.175 | 0.141 | 0.841 | 0.241 |
| | p value | 0.013 | 0.713 | 0.376 | 0.632 |
| | $\eta_p^2$ | 0.386 | 0.011 | 0.061 | 0.018 |

| | | Correct response latency | Incorrect response latency | Premature response latency | Magazine latency |
|---|---|---|---|---|---|
| **ChR2 in vHC** | | | | | |
| Laser OFF | Mean | **0.893416667** | **2.203083333** | **3.998** | **1.314** |
| | SEM | 0.038191991 | 0.235732432 | 0.153232286 | 0.095016187 |
| Laser ON | Mean | **0.989** | **2.745333333** | **4.036727273** | **1.307416667** |
| | SEM | 0.047291168 | 0.165140818 | 0.131714533 | 0.094689561 |
| | | | | | |
| **tdTomato in vHC** | | | | | |
| Laser OFF | Mean | **0.7836** | **1.133287563** | **4.073** | **1.1836** |
| | SEM | 0.024019992 | 0.506821606 | 0.095375049 | 0.036428835 |
| Laser ON | Mean | **0.7504** | **1.5354** | **4.2398** | **1.1946** |
| | SEM | 0.048171153 | 0.259181905 | 0.075960121 | 0.052013075 |
| | | | | | |
| Statistical comparison between group and conditions (Mixed ANOVA, interaction) | | | | | |
| | F value | 2.117 | 1.871 | 0.099 | 0.103 |
| | p value | 0.166 | 0.191 | 0.007 | 0.753 |
| | $\eta_p^2$ | 0.124 | 0.111 | 0.099 | 0.007 |

# Figure S4

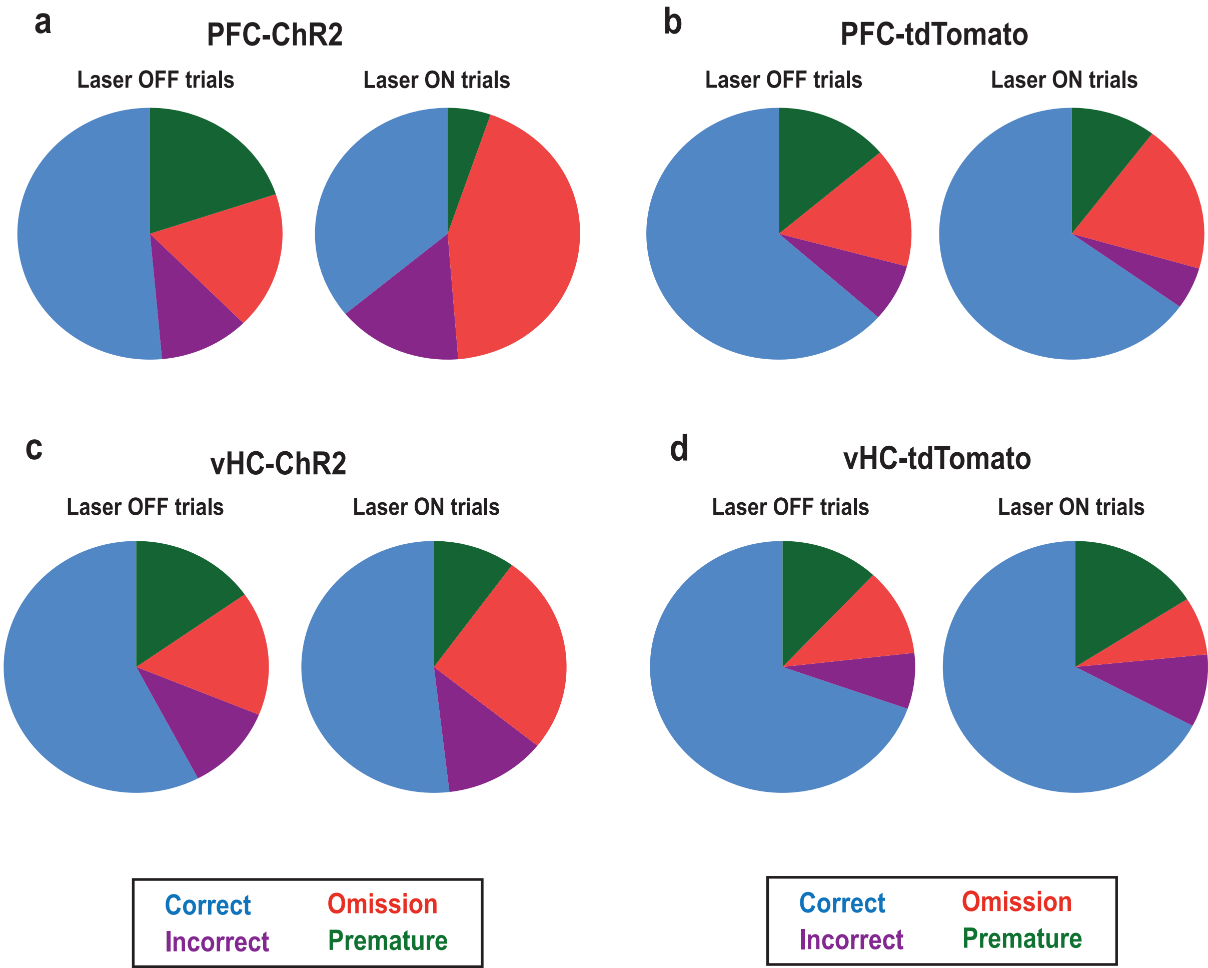
a
PFC-ChR2
Laser OFF trials
Laser ON trials
b
PFC-tdTomato
Laser OFF trials
Laser ON trials
c
vHC-ChR2
Laser OFF trials
Laser ON trials
d
vHC-tdTomato
Laser OFF trials
Laser ON trials
Correct
Omission
Incorrect
Premature
Correct
Omission
Incorrect
Premature

# Figure S5

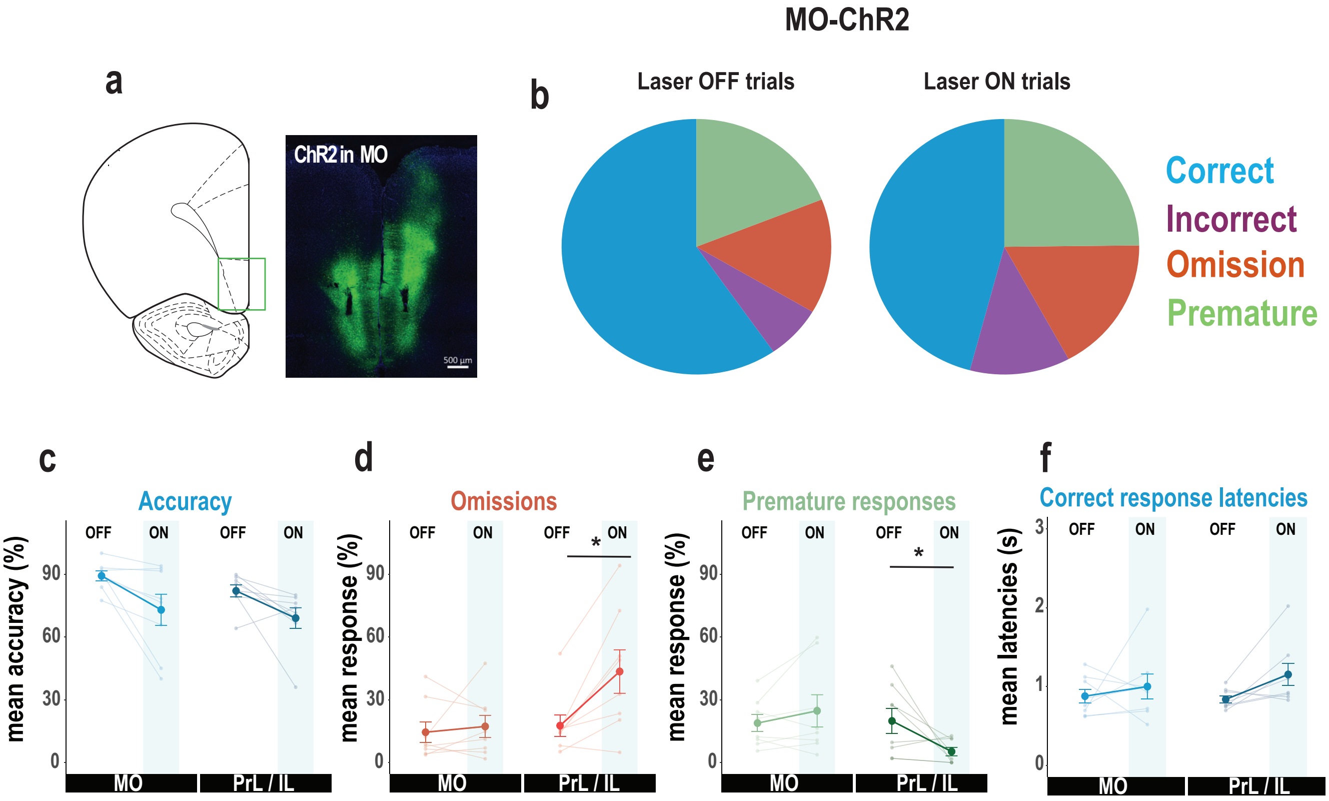
MO-ChR2
a
ChR2 in MO
500 µm
b
Laser OFF trials
Laser ON trials
Correct
Incorrect
Omission
Premature
c
Accuracy
mean accuracy (%)
OFF
ON
MO
PrL / IL
d
Omissions
mean response (%)
*
e
Premature responses
f
Correct response latencies
mean latencies (s)

# Figure S6

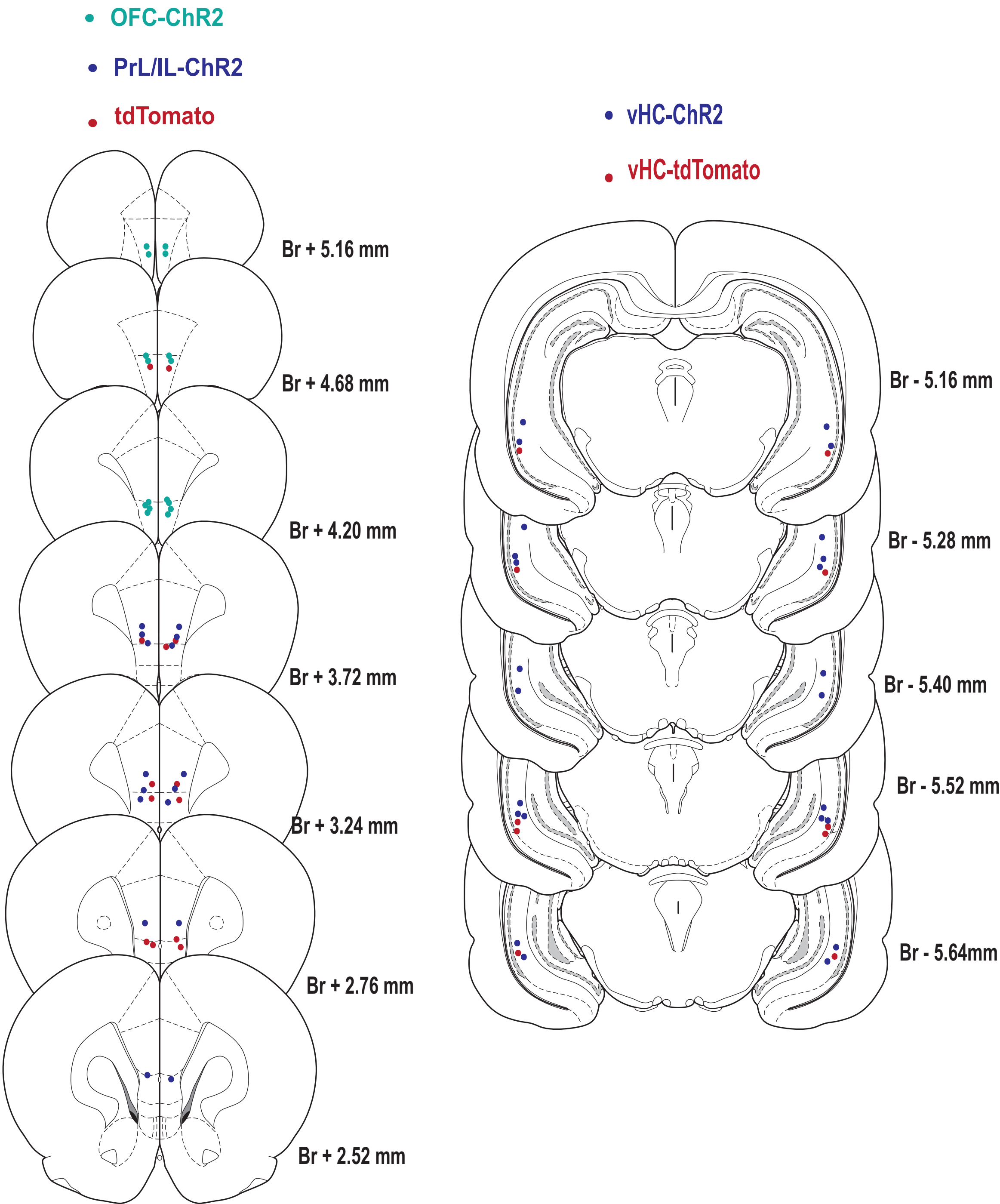
OFC-ChR2
PrL/IL-ChR2
tdTomato
vHC-ChR2
vHC-tdTomato
Br + 5.16 mm
Br + 4.68 mm
Br + 4.20 mm
Br + 3.72 mm
Br + 3.24 mm
Br + 2.76 mm
Br + 2.52 mm
Br - 5.16 mm
Br - 5.28 mm
Br - 5.40 mm
Br - 5.52 mm
Br - 5.64mm

# Figure S7

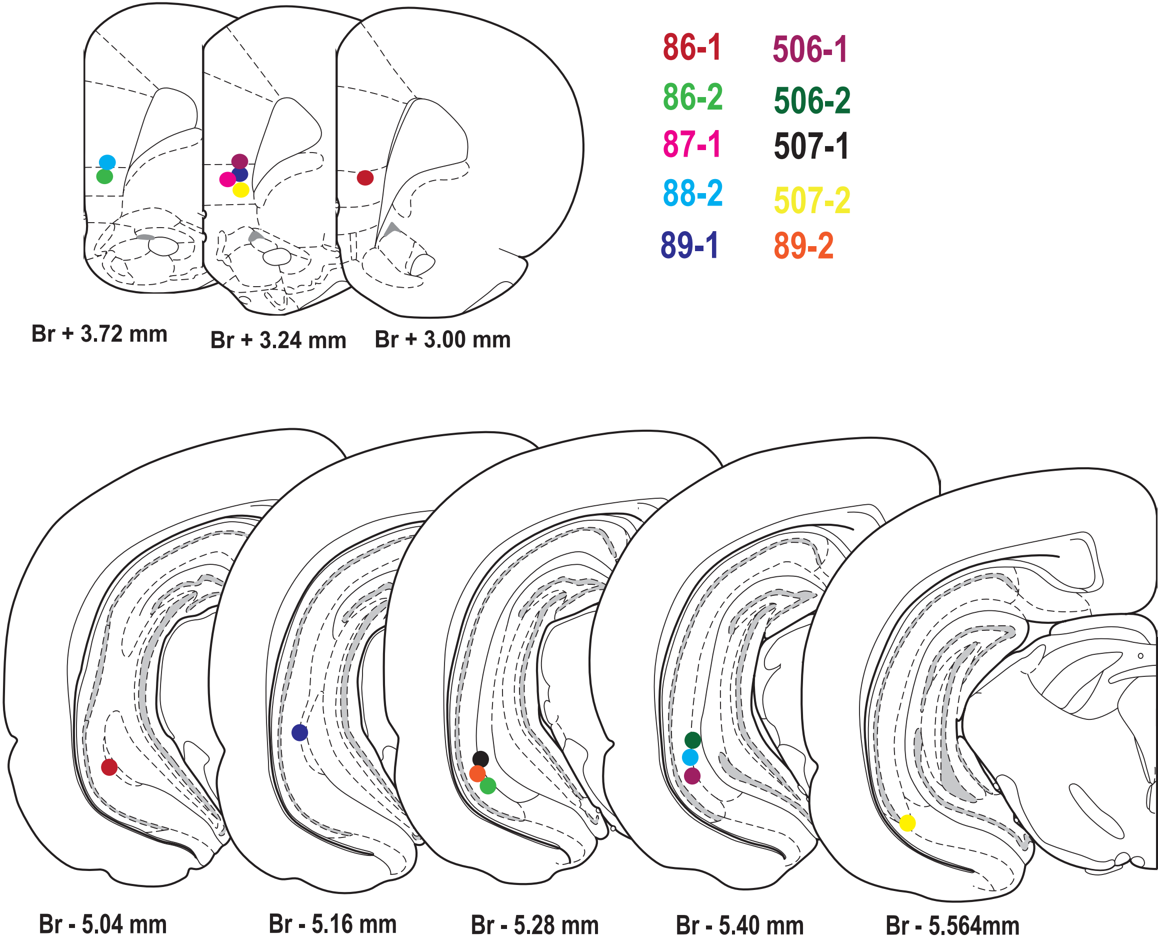
86-1
506-1
86-2
506-2
87-1
507-1
88-2
507-2
89-1
89-2
Br + 3.72 mm
Br + 3.24 mm
Br + 3.00 mm
Br - 5.04 mm
Br - 5.16 mm
Br - 5.28 mm
Br - 5.40 mm
Br - 5.564mm
