# Supplementary material for: Galanin receptor 1 expressing neurons in hippocampal-prefrontal circuitry modulate goal directed attention and impulse control
Source: bioRxiv. 2025 May 5:2024.07.29.605653. Originally published 2024 Jul 29. Preprint. [Version 2] doi: 10.1101/2024.07.29.605653 (PMC11312591; doi:10.1101/2024.07.29.605653)
Supplement: Supplementary file 1 [file media-1.pdf]

Supplementary Information

Materials and Methods

Histology and immunohistochemistry

For immunofluorescence staining, naïve animals were perfused transcardially with a working solution of PBS (1X) followed by 4% paraformaldehyde in phosphate-buffered saline. The brains were extracted and postfixed in 4% paraformaldehyde. After dehydration by immersion in 25% sucrose, the brains were cryo-sectioned at 40 μm thickness.

RNAscope in situ-hybridization (ISH)

Briefly, sections were fixated with cold 4% PFA in PBS for 1h, washed with PBS, and dehydrated in a series of increasing concentrations of ethanol in water (50%, 70%, and twice 100%). The sections were then incubated with Protease IV for 30 min and washed in distilled water. Subsequently, probes of interested were applied to the sections, and hybridization was carried on for 2h at 40 °C. This was followed by 4 amplification steps for 30 min, 15min, 30 min and 15 min respectively at 40 °C. Each amplification step was followed by two washes in Wash buffer. Sections were finally cover slipped with mounting medium containing DAPI.

Behavioral procedure: 5-choice task

Rats initiated the trial by making a nose entry into an illuminated food magazine. Following a 5 s interval, a brief white square stimulus was presented pseudo-randomly in one of five spatial locations on the screen. A correct detection within 5 s was rewarded with a single food pellet, and following a 2 s consumption time, the next trial was signaled by the illumination of the food magazine. Incorrect responses, failure to respond (omissions) or responses 'before' the stimulus presentation (premature response) terminated the trial during which the chamber was illuminated for 5 s and reward was not delivered. In addition, latencies to initiate each trial, make a response, and collect reward were recorded.

Figure S1

Expression of galanin fibers in vPFC and vHC

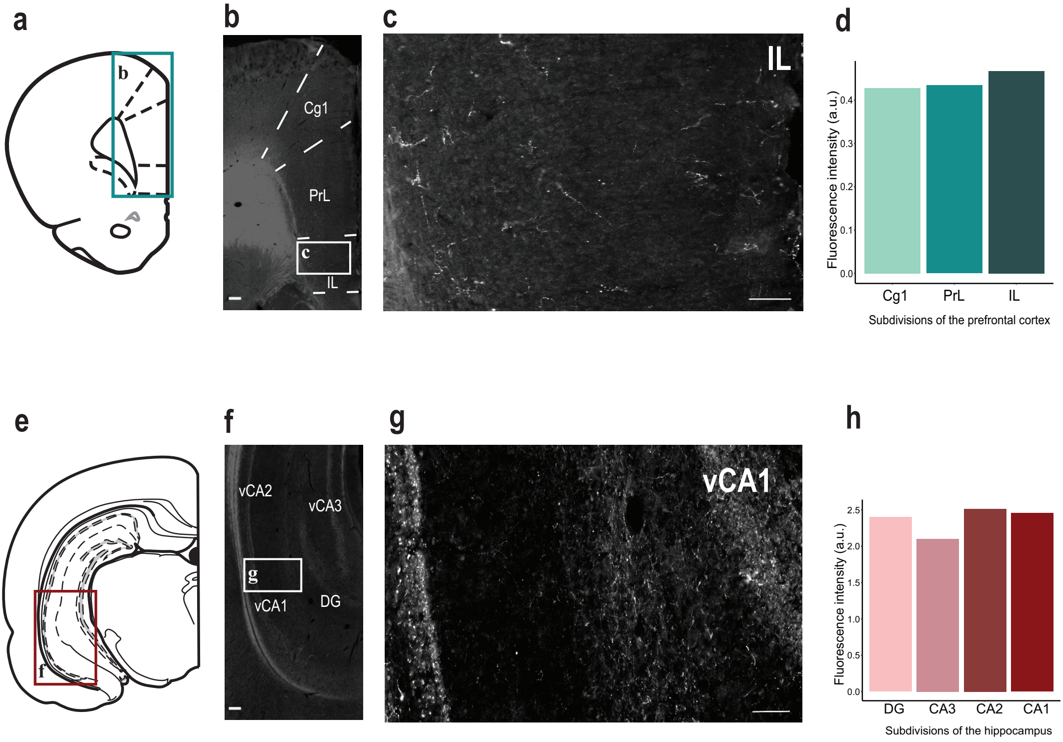

Figure S2

Statistics of the parameters measured in the 5-choice task optognetic experiments

		Accuracy	Correct response	Incorrect response	Omission	Premature response
**ChR2 in vPFC**						
Laser OFF	Mean	**82.02081**	**51.45122354**	**10.98895492**	**17.64606213**	**19.91375941**
	SEM	2.901515	5.53019645	2.003617865	5.105644181	5.989359496
Laser ON	Mean	**68.98836**	**35.98660714**	**15.28822761**	**43.48285623**	**5.242309013**
	SEM	4.986862	7.77719399	3.375468469	10.38658831	2.015927265
**tdTomato in vPFC**						
Laser OFF	Mean	**89.21182**	**63.4662332**	**7.358951268**	**15.35178713**	**13.8230284**
	SEM	2.637418	5.568235637	1.798810977	5.2688695	3.921215118
Laser ON	Mean	**91.90784**	**65.16947289**	**5.376452789**	**19.07976087**	**10.37431346**
	SEM	1.769553	5.277246771	1.187370978	4.452511768	3.314086832
Statistical comparison between groups (ChR2 and tdTomto) and conditions (Laser): (Mixed ANOVA, interaction)						
	F value	8.104	2.835	2.727	7.248	2.598
	p value	0.014	0.116	0.123	0.018	0.123
	η_p^2	0.384	0.179	0.173	0.358	0.167

		Accuracy	Correct response	Incorrect response	Omission	Premature response
ChR2 in MO						
Laser OFF	Mean	**89.1865**	**59.67896893**	**6.826730244**	**14.52529734**	**18.96900349**
	SEM	2.637418	5.307878602	1.421312652	4.927990041	4.10098683
Laser ON	Mean	**72.94635**	**45.88361308**	**12.02889976**	**17.25603009**	**24.83145707**
	SEM	7.475957	9.682159026	2.441670829	5.310827873	7.779375757
Statistical comparison between groups (PFC and MO) and conditions (Laser): (Mixed ANOVA, interaction)						
	F value	0.362	0.032	0.553	6.369	7.44
	p value	0.557	0.861	0.469	0.024	0.016
	η_p^2	0.025	0.002	0.038	0.313	0.347
Statistical comparison between groups (MO and tdTomato) and conditions (Laser): (Mixed ANOVA, interaction)						
	F value	10.592	3.855	8.878	0.078	3.509
	p value	0.006	0.71	0.011	0.784	0.084
	η_p^2	0.449	0.229	0.406	0.006	0.213

		Accuracy	Correct response	Incorrect response	Omission	Premature response
ChR2 in vHC						
Laser OFF	Mean	**85.5132**	**57.70791888**	**11.09966969**	**15.95211515**	**15.24029629**
	SEM	2.90706	3.392341087	1.876534431	2.452586439	3.240376983
Laser ON	Mean	**79.16721**	**51.86716637**	**12.37572558**	**25.74606523**	**10.01104282**
	SEM	3.49986	4.445038384	1.550673059	3.175536439	2.263969261
tdTomato in vHC						
Laser OFF	Mean	**90.70627**	**69.60419998**	**7.182922929**	**11.23568916**	**11.97718793**
	SEM	1.370147	2.739733298	1.190402456	3.272632679	0.927973488
Laser ON	Mean	**87.83151**	**67.25955986**	**9.328444508**	**7.462850442**	**15.94914519**
	SEM	2.05587	1.635296674	1.57792892	3.105930942	3.112960774
Statistical comparison between groups (ChR2 and tdTomto) and conditions (Laser): (Mixed ANOVA, interaction)						
	F value	0.023	0.303	0.04	6.672	1.939
	p value	0.882	0.59	0.844	0.021	0.184
	η_p^2	0.002	0.02	0.003	0.308	0.114

Figure S3

Statistics of the latencies measured in the 5-choice task optognetic experiments

		Correct response latency	Incorrect response latency	Premature response latency	Magazine latency
**ChR2 in vPFC**					
Laser OFF	Mean	**0.8365**	**1.942375**	**3.98875**	**1.189375**
	SEM	0.044755686	0.215749353	0.200171333	0.068890425
Laser ON	Mean	**1.15225**	**3.1695**	**3.6152**	**1.2925**
	SEM	0.141407561	0.415298946	0.228427696	0.082934785
**tdTomato in vPFC**					
Laser OFF	Mean	**0.848857143**	**1.747**	**4.165571429**	**1.118428571**
	SEM	0.07374132	0.539940164	0.161744486	0.055153836
Laser ON	Mean	**0.801857143**	**1.937666667**	**4.033833333**	**1.162142857**
	SEM	0.06668277	0.403783865	0.174455793	0.061122605
Statistical comparison between group and conditions (Mixed ANOVA, interaction)					
	F value	8.175	0.141	0.841	0.241
	p value	0.013	0.713	0.376	0.632
	η_p^2	0.386	0.011	0.061	0.018

		Correct response latency	Incorrect response latency	Premature response latency	Magazine latency
**ChR2 in vHC**					
Laser OFF	Mean	**0.893416667**	**2.203083333**	**3.998**	**1.314**
	SEM	0.038191991	0.235732432	0.153232286	0.095016187
Laser ON	Mean	**0.989**	**2.745333333**	**4.036727273**	**1.307416667**
	SEM	0.047291168	0.165140818	0.131714533	0.094689561
**tdTomato in vHC**					
Laser OFF	Mean	**0.7836**	**1.133287563**	**4.073**	**1.1836**
	SEM	0.024019992	0.506821606	0.095375049	0.036428835
Laser ON	Mean	**0.7504**	**1.5354**	**4.2398**	**1.1946**
	SEM	0.048171153	0.259181905	0.075960121	0.052013075
Statistical comparison between group and conditions (Mixed ANOVA, interaction)					
	F value	2.117	1.871	0.099	0.103
	p value	0.166	0.191	0.007	0.753
	η_p^2	0.124	0.111	0.099	0.007

Figure S4

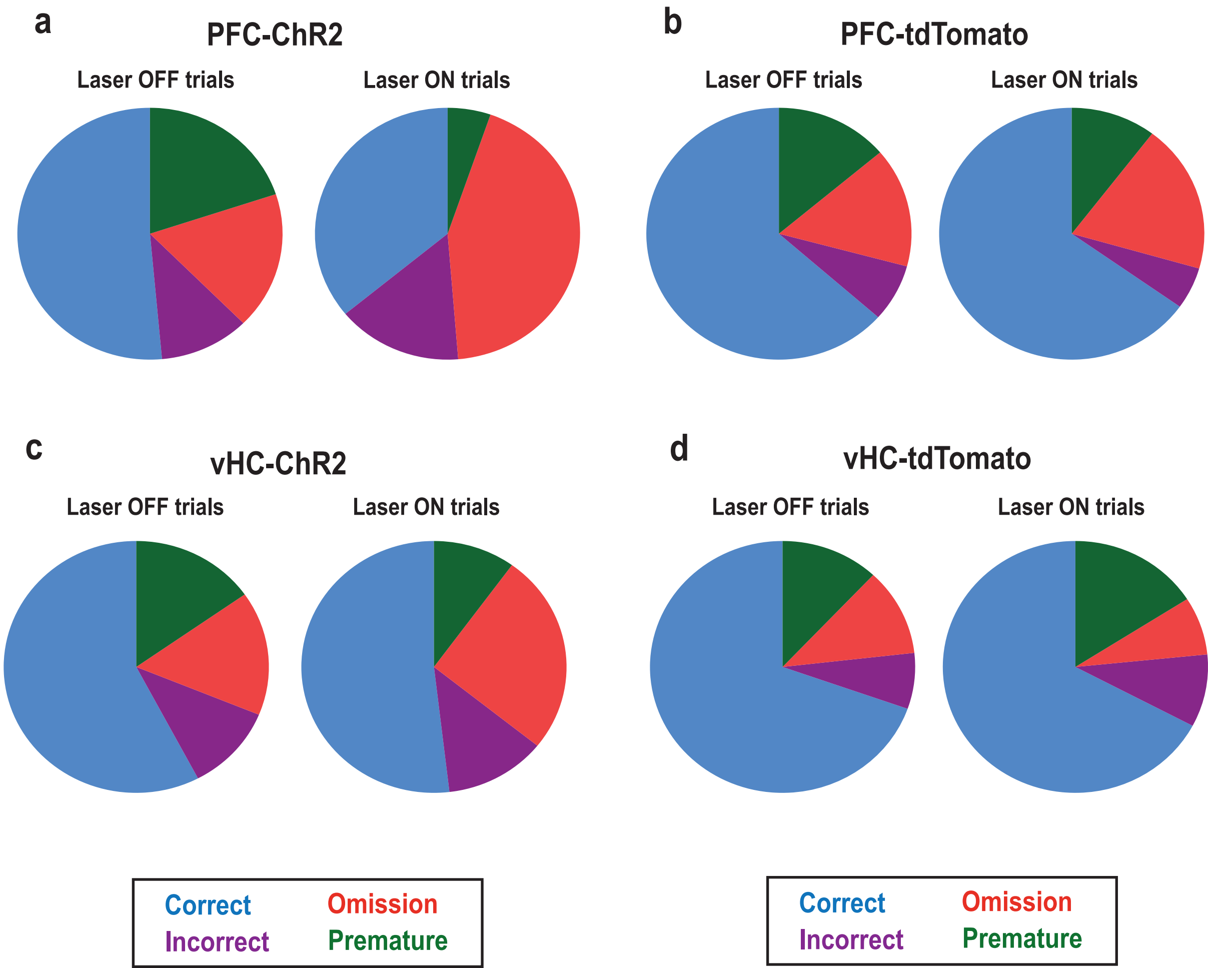
a
PFC-ChR2
Laser OFF trials
Laser ON trials
b
PFC-tdTomato
Laser OFF trials
Laser ON trials
c
vHC-ChR2
Laser OFF trials
Laser ON trials
d
vHC-tdTomato
Laser OFF trials
Laser ON trials
Correct
Omission
Incorrect
Premature
Correct
Omission
Incorrect
Premature

Figure S5

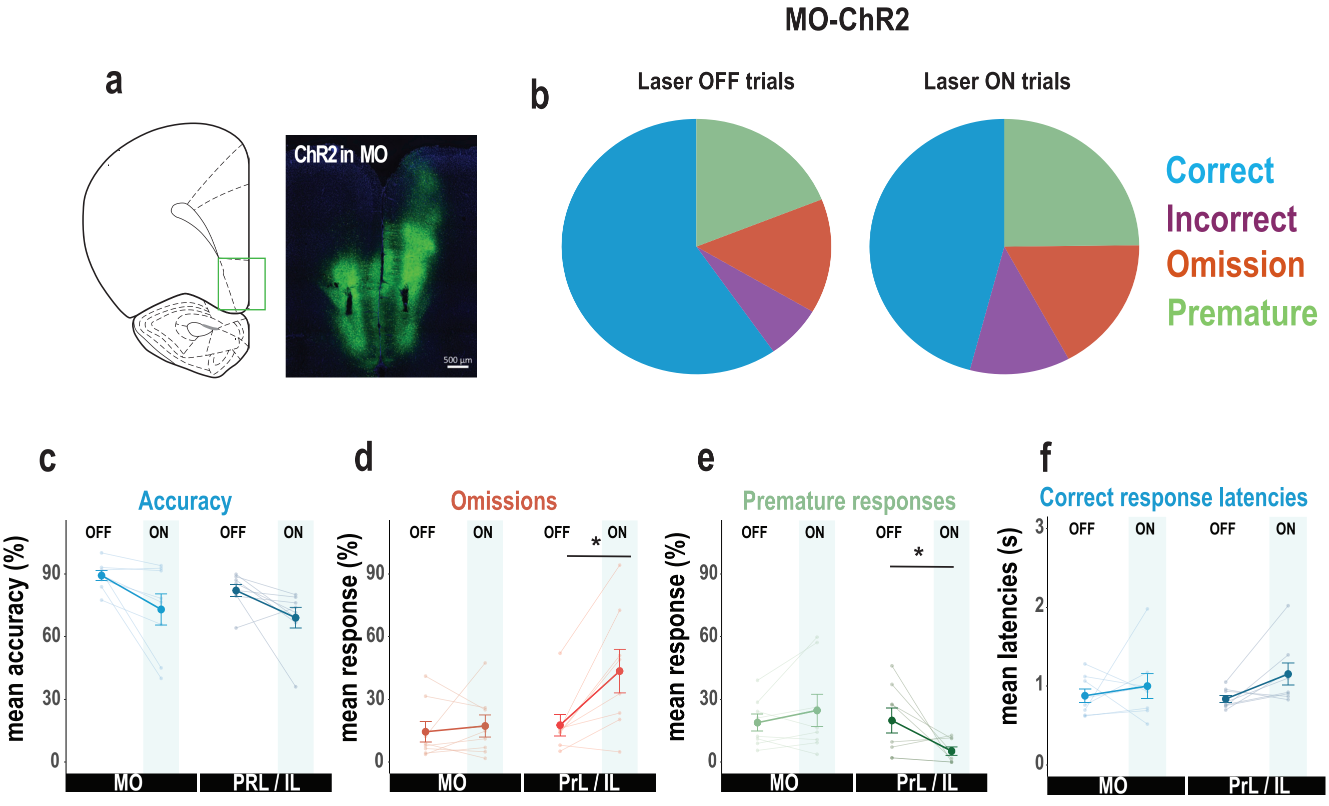

MO-ChR2
a
ChR2 in MO
500 µm
b
Laser OFF trials
Laser ON trials
Correct
Incorrect
Omission
Premature
c
Accuracy
mean accuracy (%)
OFF
ON
OFF
ON
MO
PRL / IL
d
Omissions
mean response (%)
OFF
ON
OFF
ON
*
MO
PrL / IL
e
Premature responses
mean response (%)
OFF
ON
OFF
ON
*
MO
PrL / IL
f
Correct response latencies
mean latencies (s)
OFF
ON
OFF
ON
MO
PrL / IL

Figure S6

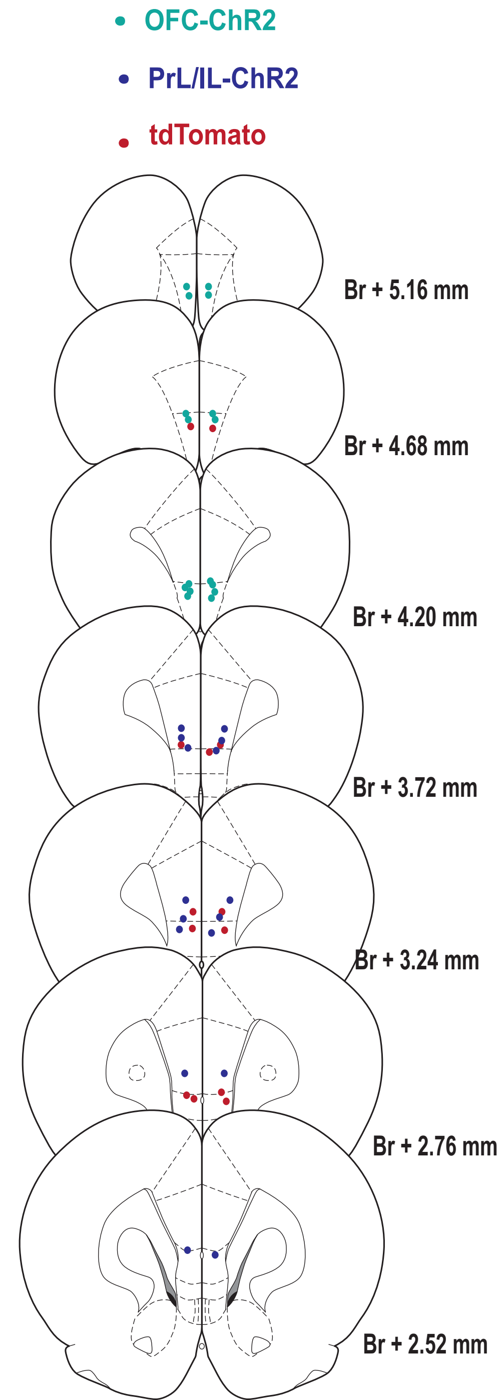
OFC-ChR2
PrL/IL-ChR2
tdTomato
Br + 5.16 mm
Br + 4.68 mm
Br + 4.20 mm
Br + 3.72 mm
Br + 3.24 mm
Br + 2.76 mm
Br + 2.52 mm

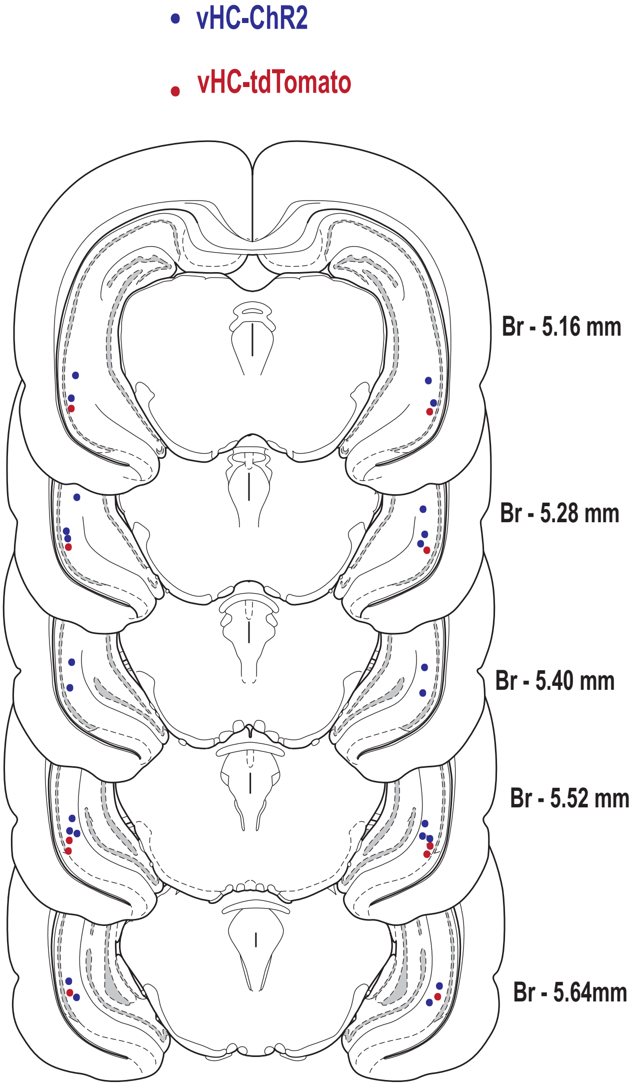
vHC-ChR2
vHC-tdTomato
Br - 5.16 mm
Br - 5.28 mm
Br - 5.40 mm
Br - 5.52 mm
Br - 5.64mm

Figure S7

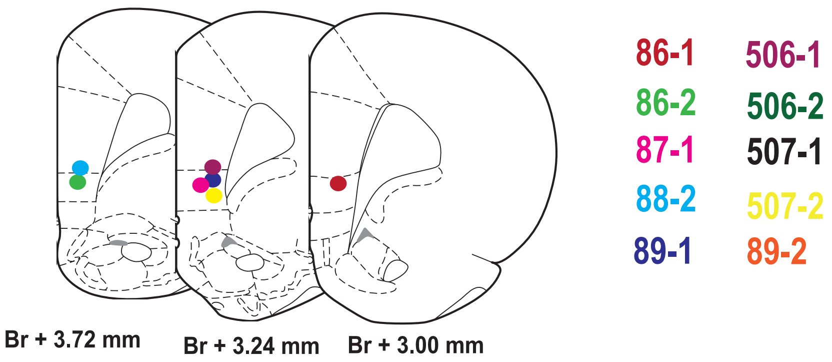
86-1
506-1
86-2
506-2
87-1
507-1
88-2
507-2
89-1
89-2
Br + 3.72 mm
Br + 3.24 mm
Br + 3.00 mm

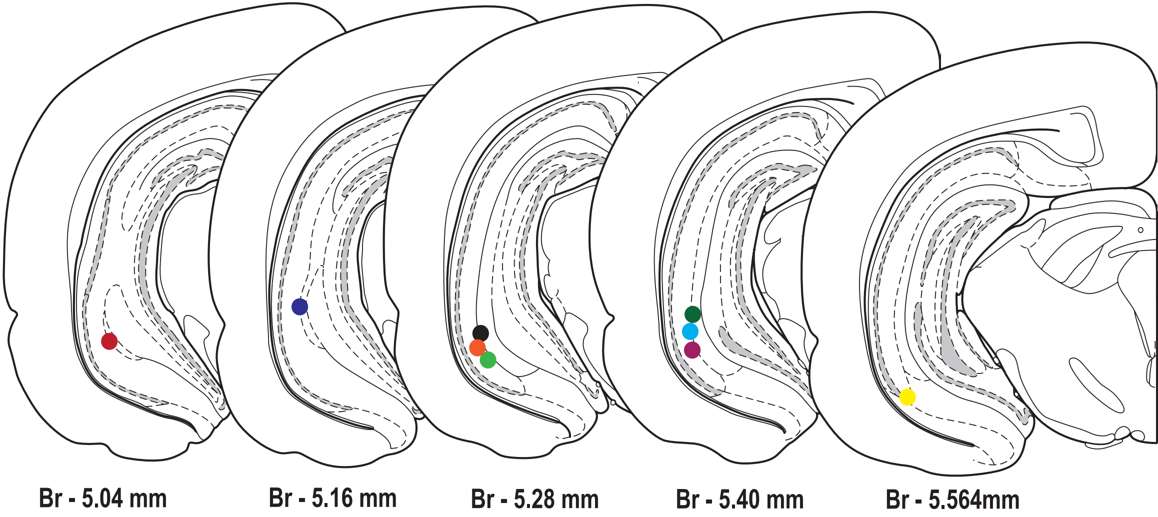
Br - 5.04 mm
Br - 5.16 mm
Br - 5.28 mm
Br - 5.40 mm
Br - 5.564mm
